# Blood culture utilization practices among febrile and/or hypothermic inpatients

**DOI:** 10.1186/s12879-022-07748-x

**Published:** 2022-10-10

**Authors:** Kap Sum Foong, Satish Munigala, Stephanie Kern-Allely, David K Warren

**Affiliations:** 1 Division of Geographic Medicine and Infectious Diseases, Tufts Medical Center, Boston, MA USA; 2 Division of Infectious Diseases, Washington University School of Medicine Hospital Epidemiologist, Barnes-Jewish Hospital, 4523 Clayton Ave., Campus Box 8051, 63110 Saint Louis, MO USA; 3 Department of Medicine, Washington University School of Medicine, St. Louis, MO USA

**Keywords:** Blood cultures, Hypothermia, Febrile illness, And diagnostic utility

## Abstract

**Background:**

Predictors associated with the decision of blood culture ordering among hospitalized patients with abnormal body temperature are still underexplored, particularly non-clinical factors. In this study, we evaluated the factors affecting blood culture ordering in febrile and hypothermic inpatients.

**Methods:**

We performed a retrospective study of 15,788 adult inpatients with fever (≥ 38.3℃) or hypothermia (< 36.0℃) from January 2016 to December 2017. We evaluated the proportion of febrile and hypothermic episodes with an associated blood culture performed within 24h. Generalized Estimating Equations were used to determine independent predictors associated with blood culture ordering among febrile and hypothermic inpatients.

**Results:**

We identified 21,383 abnormal body temperature episodes among 15,788 inpatients (13,093 febrile and 8,290 hypothermic episodes). Blood cultures were performed in 36.7% (7,850/ 21,383) of these episodes. Predictors for blood culture ordering among inpatients with abnormal body temperature included fever ≥ 39℃ (adjusted odd ratio [aOR] 4.17, 95% confident interval [CI] 3.91–4.46), fever (aOR 3.48, 95% CI 3.27–3.69), presence of a central venous catheter (aOR 1.36, 95% CI 1.30–1.43), systemic inflammatory response (SIRS) plus hypotension (aOR 1.33, 95% CI 1.26–1.40), SIRS (aOR 1.26, 95% CI 1.20–1.31), admission to stem cell transplant / medical oncology services (aOR 1.09, 95% CI 1.04–1.14), and detection of abnormal body temperature during night shift (aOR 1.06, 95% CI 1.03–1.09) or on the weekend (aOR 1.05, 95% CI 1.01–1.08).

**Conclusion:**

Blood culture ordering for hospitalized patients with fever or hypothermia is multifactorial; both clinical and non-clinical factors. These wide variations and gaps in practices suggest opportunities to improve utilization patterns.

**Supplementary Information:**

The online version contains supplementary material available at 10.1186/s12879-022-07748-x.

## Introduction

Fever is a common finding among hospitalized patients, occurring in 2 to 29% of hospital stays. [1–3] While fever is often a clinical indication for performing blood culture, there is a poor correlation between fever and bloodstream infections. [4–6] The reported incidence of positive blood cultures among hospitalized febrile patients is 5–15%; as many as 50% of these positive cultures are due to contamination. [4, 5, 7, 8] False positive blood cultures are associated with exposure to unnecessary antibiotics, increased downstream testing, healthcare cost, and hospital length of stay. [9–11]

Recent work suggests that blood-culturing practices are varied by clinical services, provider’s role, and years of clinical experience. [12, 13] However, predictors associated with the decision of blood culture ordering among hospitalized patients with abnormal body temperature are still underexplored, particularly non-clinical factors. While fever has been the focus of prior research, much less is known about the utilization pattern of blood cultures among hospitalized patients with hypothermia.

In this study, our objective was to determine the factors affecting blood culture ordering in febrile and hypothermic inpatients. Given the increasing focus on diagnostic stewardship, a better understanding of factors associated with blood culture performance among hospitalized patients with abnormal body temperature could help efforts to reduce inappropriate and unnecessary testing.

### Methods

#### Design and sample

We conducted a retrospective analysis of all hospitalized adults aged 18 years and older who had at least one episode of fever or hypothermia at a 1,250 bed tertiary academic medical center between January 2016 and December 2017. Patients included in the study were admitted to the hospital for a variety of reasons. Patients who were on hypothermia protocol (i.e., post-cardiac arrest management) were excluded. Pertinent data on patient’s demographics, comorbidities (using International Classification of Diseases, 10th revision, eTable 1), hospital encounter, vital signs, blood cultures, mechanical ventilation, presence of central venous catheter, and indwelling urinary catheter were extracted from hospital medical informatics database.

#### Study definitions

Fever and Hypothermia: We defined clinically significant fever as a single recorded body temperature of ≥ 38.3℃ (101.0℉). [14] Hypothermia was defined as a single recorded body temperature of < 36.0℃ (96.8℉). [14] Since patients could have prolonged or more than one episode of fever or hypothermia per hospital admission, we defined a new febrile or hypothermic episode as re-occurrence of fever or hypothermia, after being normothermic for ≥ 48h. [1] If a patient had hypothermia and hyperthermia measurements within 24h, only the highest temperature recorded during that period is considered to classify the episode. At our institution, temperature measurements were obtained from central site (e.g., pulmonary artery, rectal, or urinary bladder,) or peripheral site (e.g., tympanic, temporal or axillary) every 4h at a minimum, but this can be varied based on acuity of illness and at the discretion of the healthcare personnel.

Systemic Inflammatory Response Syndrome (SIRS): We evaluated the presence of SIRS within 24h of the onset of each episode of fever or hypothermia. In addition to an abnormal body temperature (≥ 38.3℃ or < 36℃), SIRS was defined as the presence of at least one of the following three criteria; (1) heart rate > 90 beats/ minutes; (2) respiratory rate > 20 breaths/ minutes; or (3) white blood count > 12,000 or < 4,000 cells/mm^3^. [15, 16] Based on previous literature, we further defined SIRS plus hypotension as presence of a systolic blood pressure < 90 mmHg within 24h of a SIRS event. [16]

Blood culture: During the study period, our institution utilized the VersaTREK blood culture system (Thermo Scientific, Waltham, MA) for blood cultures. A single blood culture was comprised of two separate bottles (VersaTREK REDOX 1 [aerobic media] and REDOX2 [anaerobic media]). A positive blood culture was considered as contaminated if a common commensal (e.g., diphtheroids, *Bacillus* spp., *Propionibacterium* spp., coagulase-negative staphylococci, micrococci, etc.) was isolated in a single blood specimen, using National Healthcare Safety Network criteria. [17] We defined a true-positive blood culture as the isolation of an organism generally regarded as pathogenic, or a common commensal growing in more than one set of blood cultures. [13, 18] For the purpose of this study, only blood cultures obtained within one calendar day of fever or hypothermia episodes were considered.

Other definitions. We defined 8 am to 8 pm as a day shift and 8 pm to 8 am of the following day as a night shift. A hospital-onset fever or hypothermia was defined as an abnormal body temperature episode occurring on hospital day three or later.

True positive blood cultures rate: number of hypothermic or fever episodes with true positive blood cultures by the total numbers of hypothermic or fever episodes.

Diagnostic yield of blood cultures: number of hypothermic or fever episodes with true positive blood cultures by the numbers of hypothermic or fever episodes with blood cultures obtained.

#### Outcome measures

The primary outcome was to identify predictors associated with blood culture ordering among adult inpatients with febrile or hypothermic episodes. The secondary outcome was to evaluate the proportion of true-positive and contaminant blood cultures.

### Statistical analysis

Demographic and baseline clinical characteristics of patients were described in number (percentage) for categorical variables and median (interquartile range [IQR]) for continuous variables.

For the evaluation of primary and secondary outcomes, we used per-episode of fever or hypothermia as the unit of analysis. Descriptive analysis of baseline clinical characteristics and risk factors were compared (i) between febrile and hypothermic episodes and (ii) based on blood culture ordering status using Chi square, Fisher exact test, or univariate logistic regression for categorical variables, and Kruskal-Wallis test for continuous variables. Generalized Estimating Equations (GEE) using the REPEATED statement on PROC GENMOD were utilized for the multivariable analysis to determine independent predictors associated with blood culture ordering among febrile and hypothermic adult inpatients. Since each patient may have had more than one episode of fever or hypothermia within the same admission, we used GEE method to account for multiple episodes within the same admission. To evaluate the role of extreme hyperthermia (temperature ≥ 39℃) on blood culture ordering practices, we further divided febrile episodes to extreme hyperthermia group and the rest of febrile episodes < 39℃. Hypothermic episodes without obtainment of associated blood culture were regarded as the reference or control group. All variables with *p* < .20 in univariate analysis were considered for entry in GEE model. All statistical tests were two-tailed and significance was set at α = 0.05. Data were analyzed using SAS version 9.4 Software (SAS Institute Inc., Cary, NC).

## Results

### Characteristics

During the study period, we identified a total of 21,383 episodes of abnormal body temperature among 15,788 hospitalized patients. There were 13,093 (61.2%) febrile and 8,290 (38.8%) hypothermic episodes; representing an overall incidence rate of 20.8 episodes of fever and 13.2 episodes of hypothermia per 1,000 patient-days. The median age of patients was 59 years (IQR, 46 to 69 years), 55.4% were male, 65.6% were white, and 58.8% were hospital-onset. The median hospital length of stay was 12 days (IQR, 6 to 26 days). Majority of these patients had their abnormal body temperature detected during day shifts (57.8%) and on the weekdays (74.2%) (Table 1).

**Table 1:** Comparison of 21,383 episodes of abnormal temperature (fever vs. hypothermia) among 15,788 adult inpatients

Variable	Total n = 21,383^α^; n (%)	Febrile episodes n = 13,093; n (%)	Hypothermic episodes n = 8290; n (%)	*p*-value
Age in years, median (IQR)	59 (46–69)	57 (42–67)	62 (51–71)	< 0.0001
Gender				0.218
Male	11,841 (55.4)	7294 (55.7)	4547 (54.8)	
Female	9542 (44.6)	5799 (44.3)	3743 (45.2)	
Race				0.350
White	14,036 (65.6)	8626 (65.9)	5410 (65.3)	
Black and others	7347 (34.4)	4467 (34.1)	2880 (34.7)	
Hospital-onset	12,563 (58.8)	7656 (58.5)	4907 (59.2)	0.299
Shift work during detection of abnormal body temperature				< 0.0001
Day (8am to 8pm)	12,349 (57.8)	7279 (55.6)	5070 (61.2)	
Night (8pm to 8am of the following day)	9034 (42.2)	5814 (44.4)	3220 (38.8)	
Weekend (Saturday & Sunday)	5512 (25.8)	3599 (27.5)	1913 (23.1)	< 0.0001
Mechanical ventilation	8887 (41.6)	4900 (37.4)	3987 (48.1)	< 0.0001
Indwelling urinary catheter	13,991 (65.4)	8040 (61.4)	5951 (71.8)	< 0.0001
Central venous catheter	14,367 (67.2)	8856 (67.6)	5511 (66.5)	0.078
Comorbidities				
Congestive heart failure	5873 (27.5)	2801 (21.4)	3072 (37.1)	< 0.0001
Chronic lung disease	5101 (23.9)	2824 (21.6)	2277 (27.5)	< 0.0001
Diabetes mellitus	5866 (27.4)	3282 (25.1)	2584 (31.2)	< 0.0001
Chronic liver failure	3465 (16.2)	1720 (13.1)	1745 (21.0)	< 0.0001
End stage renal disease	5029 (23.5)	2393 (18.3)	2636 (31.8)	< 0.0001
Malignancy	2852 (13.3)	1721 (13.1)	1131 (13.6)	0.296
HIV infection	256 (1.2)	178 (1.4)	78 (0.9)	0.007
SIRS status				
No SIRS	6185 (28.9)	3975 (30.4)	2210 (26.7)	Reference
SIRS alone	4467 (20.9)	3187 (24.3)	1280 (15.4)	< 0.0001
SIRS plus hypotension	10,731 (50.2)	5931 (45.3)	4800 (57.9)	< 0.0001
WBC				
Normal or not done	7282 (34.1)	3862 (29.5)	3420 (41.3)	Reference
<4,000 /mm^3^	3713 (17.3)	2801 (21.4)	912 (11.0)	< 0.0001
>12,000 /mm^3^	10,388 (48.6)	6430 (49.1)	3958 (47.7)	< 0.0001
Associated blood culture obtained (within one calendar day)	7850 (36.7)	6758 (51.6)	1092 (13.2)	< 0.0001
Positive blood cultures				
True positive	672 (3.1)	603 (4.6)	69 (0.8)	0.004
Contamination^β^	44 (0.2)	38 (0.3)	6 (0.1)	1.00
Department during detection of abnormal body temperature				
Emergency department	648 (3.0)	256 (2.0)	392 (4.7)	< 0.0001
General medicine	2854 (13.3)	2042 (15.6)	812 (9.8)	Reference
Surgery	2669 (12.4)	1853 (14.2)	816 (9.9)	0.084
ICU	10,127 (47.4)	5361 (40.9)	4766 (57.5)	< 0.0001
SCT / medical oncology	2565 (12.0)	2240 (17.1)	325 (3.9)	< 0.0001
Cardiology	359 (1.7)	143 (1.1)	216 (2.6)	< 0.0001
Neurology / neurosurgery	623 (2.9)	359 (2.7)	264 (3.2)	< 0.0001
Gynecologic oncology	226 (1.1)	191 (1.5)	35 (0.4)	< 0.0001
Obstetrics	421 (2.0)	280 (2.1)	141 (1.7)	0.034
Orthopedics	341 (1.6)	219 (1.7)	122 (1.5)	0.005
Psychiatry	421 (2.0)	80 (0.6)	341 (4.1)	< 0.0001
Others	129 (0.6)	69 (0.5)	60 (0.7)	< 0.0001
Length of stay in days, median (IQR)	12 (6–26)	12 (6–27)	12 (5–26)	0.952
Discharge status^γ^				
Home	12,176 (56.9)	7995 (61.1)	4181 (50.5)	Reference
Facility^µ^	6020 (28.2)	3663 (28.0)	2357 (28.4)	< 0.0001
In-hospital death	3183 (14.9)	1431 (10.9)	1752 (21.1)	< 0.0001

ICU, intensive care unit; IQR, interquartile range; HIV, human immunodeficiency virus; SCT, stem cell transplant; SIRS, systemic inflammatory response syndrome; WBC, white blood count

^α^Number of fever or hypothermia episodes per admission (median 1; IQR 1 to 2; minimum 1 and maximum 14)

^β^46 organisms were isolated from 44 contaminant blood cultures

^γ^Missing 4 (hyperthermia-4; hypothermia-0)

^µ^Includes skilled nursing facility (n = 3,106), other facility (n = 2,909), and remained in the hospital at the end of study period (n = 5)

Patients in the hypothermic group were older (median, 62 vs. 57 years, *p* < .0001), more likely to have an abnormal body temperature detected during day shifts (61.2% vs. 55.6%, *p* < .0001), on mechanical ventilation (48.1% vs. 37.4%, *p* < .0001), presence of indwelling urinary catheter (71.8% vs. 61.4%, *p* < .0001), SIRS plus hypotension (57.9% vs. 45.3%, *p* < .0001), and admission to intensive care unit (ICU) (57.5% vs. 40.9%, *p* < .0001) compared to the febrile group. Comorbidities were also more frequent in the hypothermic patients, other than human immunodeficiency virus infection and malignancy. Distribution of gender and the presence of central venous catheter were similar between the two groups (Table 1).

There were more patients who had SIRS (24.3% vs. 15.4%, *p* < .0001), WBC < 4,000 cells/mm^3^ (21.4% vs. 11.0%, *p* < .0001), WBC > 12,000 cells/mm^3^ (49.1% vs. 47.7%, *p* < .0001), and admission to stem cell transplant (SCT) / medical oncology services (17.1% vs. 3.9%, *p* < .0001) in the febrile group. The all-cause in-hospital mortality rate was higher among patients with hypothermia than those with febrile episodes (21.1% vs. 10.9%, *p* < .0001).

### Diurnal variations in fever and hypothermia

**Fig. 1:**
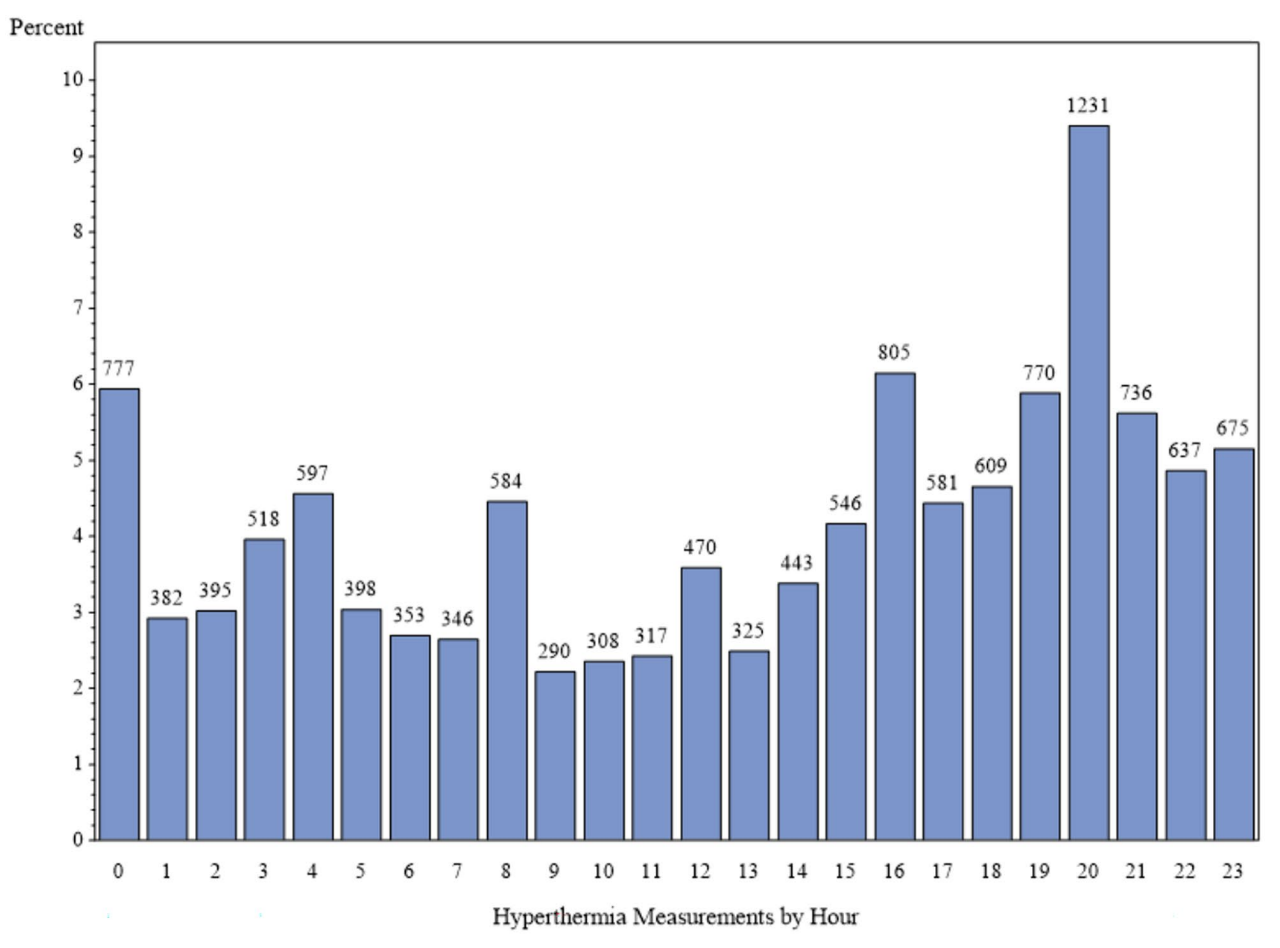
Diurnal variation during the initial measurement of abnormal temperature for 13,093 episodes of fever. Y-axis- percentage of febrile/hypothermic episodes by hour

**Fig. 2:**
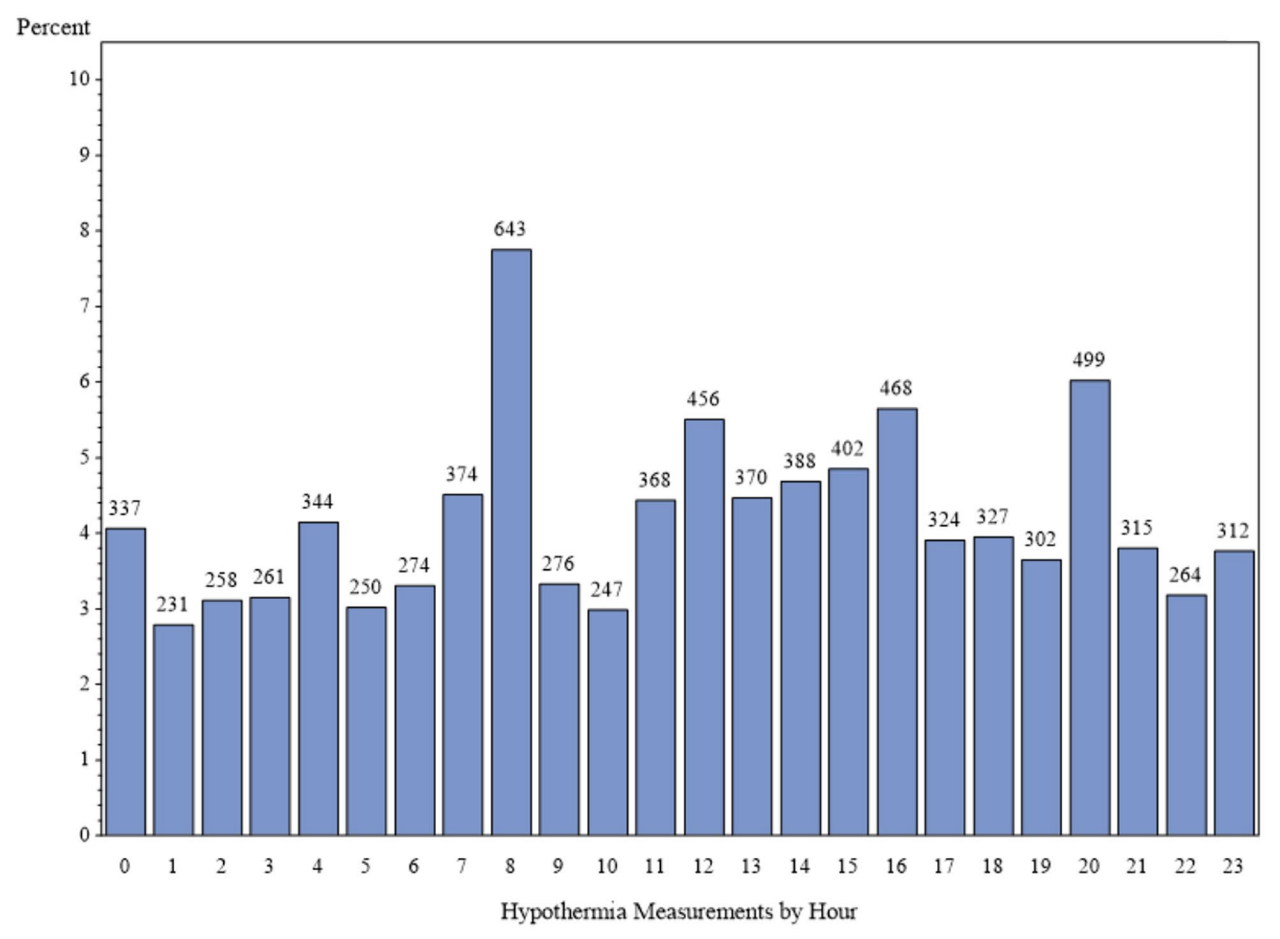
Diurnal variation during the initial measurement of abnormal temperature for 8,290 episodes of hypothermia. Y-axis- percentage of hypothermic episodes by hour

**Fig. 3:**
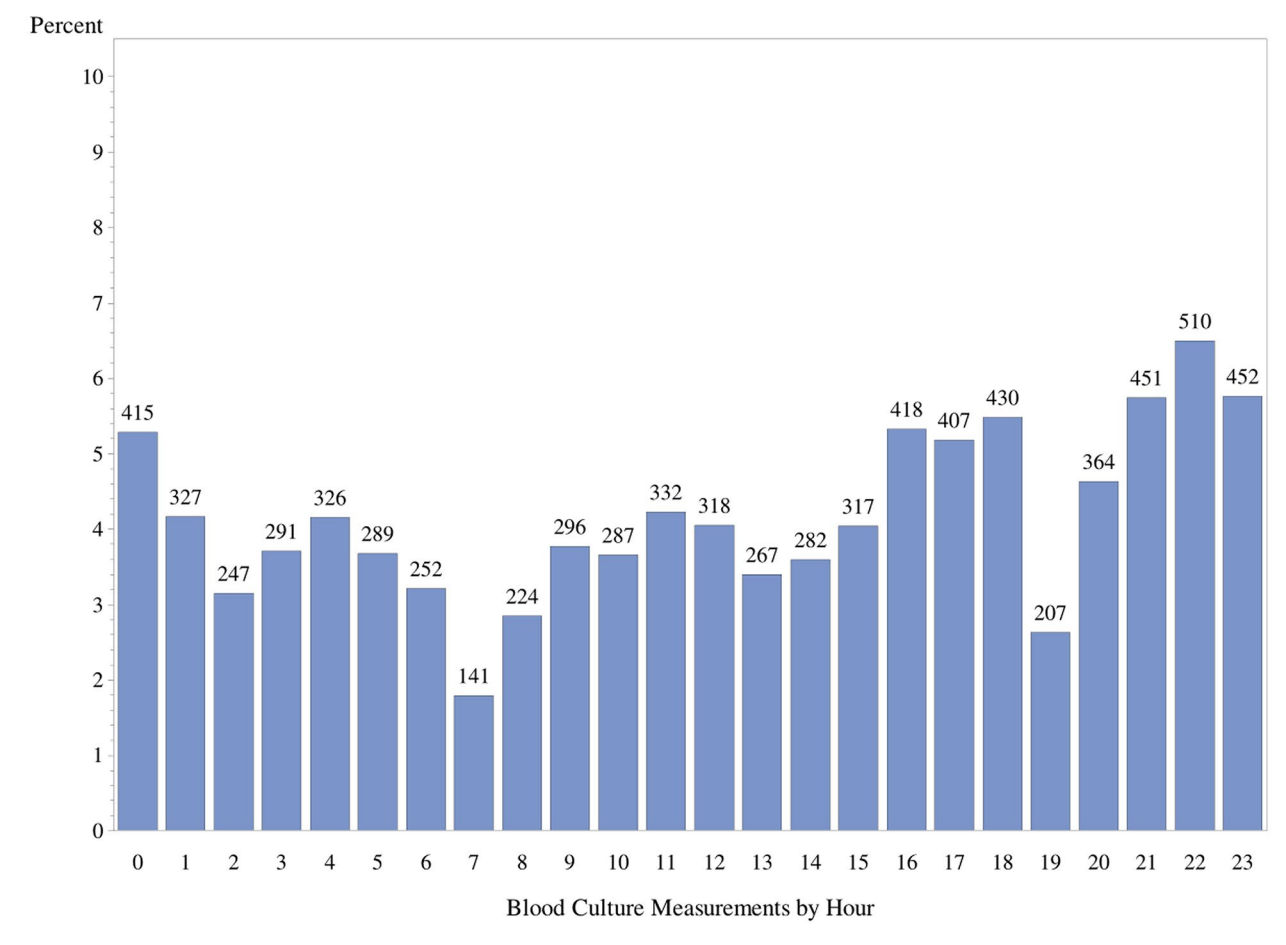
Diurnal variation in 7,850 blood cultures obtained for 21,390 episodes of fever and hypothermia. Y-axis- percentage of febrile episodes by hour

As shown in Figs. 1 and 2, there was diurnal variation of fever and hypothermia with detection of multiple peaks virtually identical between the two groups at time 01:00, 04:00, 08:00, 12:00, 16:00, and 20:00. We further examined diurnal pattern of the number of blood cultures performed and found no temporal association with these peaks of abnormal body temperature (Fig. 3).

### Blood-culture ordering practices for febrile and hypothermic inpatients

Of 21,383 episodes of abnormal body temperature, 7,850 (36.7%) episodes had one or more associated blood cultures performed. Febrile inpatients were more likely to have at least one associated blood cultures obtained compared to those with hypothermia (6,758 [51.6%] vs. 1,902 [13.2%], *p* < .0001).

Overall, blood cultures were true positive in 3.1% (n = 672) with a diagnostic yield of 8.5%. This true-positive blood culture rate was significantly higher in the febrile group compared to the hypothermic group (603 [4.6%] vs. 69 [0.8%], *p* = .004). We observed no difference in the rate of blood culture contamination between the two groups (0.3% in febrile group vs. 0.1% hypothermic group, *p* = 1.00) (Table 1).

The most commonly isolated pathogens were *Staphylococcus aureus* (22.0%), *Enterobacterales* (17.8%), coagulase negative *Staphylococcus spp.* (16.3%), enterococci (10.2%), and *Candida* spp. (7.0%). The proportion of coagulase negative *Staphylococcus* spp. was significantly higher in hypothermic group. All of the *Streptococcus* spp. isolated were from patients with hyperthermia. No other significant differences were observed in the remaining identified organisms. The full list of isolated pathogens is shown in eTable 2.

### Predictors for blood-culturing ordering practices

Univariate and multivariable analyses for predictors associated with blood culture ordering are summarized on Table 2. On multivariable analysis, fever ≥ 39℃ (adjusted odd ratio [aOR] 4.17, 95% confident interval [CI] 3.91 to 4.46), fever (aOR 3.48, 95% CI 3.27 to 3.69), presence of SIRS plus hypotension (aOR 1.32, 95% CI 1.25 to 1.39), SIRS (aOR, 1.25; 95% CI, 1.19 to 1.31), central venous catheter (aOR 1.36, 95% CI 1.30 to 1.43), chronic liver failure (aOR 1.18, 95% CI 1.13 to 1.23), detection of abnormal body temperature during night shift (aOR 1.06, 95% CI 1.03 to 1.09) and on the weekend (aOR 1.05, 95% CI 1.01 to 1.09), and admission to SCT / medical oncology services (aOR 1.09, 95% CI 1.04 to 1.15) were significant predictors for blood culture ordering among hospitalized patients with at least one episode of abnormal body temperature. In the same analysis, factors associated with less blood culturing included presence of indwelling urinary catheter (aOR 0.93, 95% CI 0.89 to 0.97), WBC > 12,000/mm^3^ (aOR 0.90, 95% CI 0.86 to 0.94), and admission to ICU (aOR 0.69, 95% CI 0.65 to 0.73) or surgery (aOR 0.56, 95% CI 0.52 to 0.60).

**Table 2:** Comparison of factors for blood culture ordering among febrile or hypothermic adult inpatients

Variable	Blood culture performed n = 7850; n (%)	No blood culture performed n = 13,533; n (%)	Univariate analysis	Multivariable analysis ^α^	
			***p*** **value**	**aOR** **(95% CI)**	***p*** **value**
Age in years, median (IQR)	59 (46–68)	60 (45–69)	< 0.0001	-	
Gender			0.69	-	
Male	4333 (55.2)	7508 (55.5)			
Female	3517 (44.8)	6025 (44.5)			
Race			0.003		
White	5254 (66.9)	8782 (64.9)		Reference	
Black or others	2596 (33.1)	4751 (35.1)		1.07 (1.03–1.11)	0.001
Type of abnormal body temperature					
Fever ≥ 39℃	1664 (21.2)	1035 (7.7)	< 0.0001	4.17 (3.91–4.46)	< 0.0001
Fever	5094 (64.9)	5300 (39.1)	< 0.0001	3.48 (3.27–3.69)	< 0.0001
Hypothermia	1092 (13.9)	7198 (53.2)	Reference	Reference	
Hospital-onset fever or hypothermia	4857 (61.9)	7706 (56.9)	< 0.0001	1.02 (0.98–1.06)	0.23
Comorbidities					
Congestive heart failure	1893 (24.1)	3980 (29.4)	< 0.0001	0.97 (0.93–1.02)	0.244
Chronic lung disease	1735 (22.1)	3366 (24.9)	< 0.0001	1.01 (0.97–1.05)	0.654
Diabetes mellitus	2061 (26.3)	3805 (28.1)	0.003	1.01 (0.97–1.04)	0.754
Chronic liver failure	1354 (17.2)	2111 (15.6)	0.002	1.18 (1.13–1.23)	< 0.0001
End stage renal disease	1672 (21.3)	3357 (24.8)	< 0.0001	1.03 (0.98–1.07)	0.189
Malignancy	1053 (13.4)	1799 (13.3)	0.803	-	
HIV infection	114 (1.5)	142 (1.0)	0.010	1.03 (0.90–1.16)	0.754
SIRS status					
No SIRS	1926 (24.5)	4259 (31.5)	Reference	Reference	
SIRS alone	1977 (25.2)	2490 (18.4)	< 0.0001	1.25 (1.19–1.31)	< 0.0001
SIRS plus hypotension	3947 (50.3)	6784 (50.1)	< 0.0001	1.32 (1.25–1.39)	< 0.0001
WBC					
Normal or not done	2197 (28.0)	5085 (37.6)	Reference	Reference	
<4,000 /mm^3^	2194 (27.9)	1519 (11.2)	< 0.0001	1.03 (0.98–1.08)	0.292
>12,000 /mm^3^	3459 (44.1)	6929 (51.2)	< 0.0001	0.90 (0.86–0.94)	< 0.0001
Shift work during detection of abnormal body temperature			< 0.0001		
Day (8am to 8pm)	4286 (54.6)	8063 (59.6)		Reference	
Night (8pm to 8am of the following day)	3564 (45.4)	5470 (40.4)		1.06 (1.03–1.09)	< 0.0001
Weekend (Saturday & Sunday)	2217 (28.2)	3295 (24.3)	< 0.0001	1.05 (1.01–1.09)	0.005
Mechanical ventilation	3222 (41.0)	5665 (41.9)	0.243	-	
Indwelling urinary catheter	4784 (60.9)	9207 (68.0)	< 0.0001	0.93 (0.89–0.97)	0.001
Central venous catheter	6110 (77.8)	8257 (61.0)	< 0.0001	1.36 (1.30–1.43)	< 0.0001
Department during detection of abnormal body temperature					
Emergency department	233 (3.0)	415 (3.1)	< 0.0001	0.99 (0.89–1.10)	0.894
General medicine	1391 (17.7)	1463 (10.8)	Reference	Reference	
Surgery	677 (8.6)	1992 (14.7)	< 0.0001	0.56 (0.52–0.60)	< 0.0001
ICU	3167 (40.3)	6960 (51.4)	< 0.0001	0.69 (0.65–0.73)	< 0.0001
SCT / medical oncology	1850 (23.6)	715 (5.3)	< 0.0001	1.09 (1.04–1.15)	0.002
Cardiology	97 (1.2)	262 (1.9)	< 0.0001	0.73 (0.63–0.85)	< 0.0001
Neurology / neurosurgery	173 (2.2)	450 (3.3)	< 0.0001	0.72 (0.69–0.81)	< 0.0001
Gynecologic oncology	126 (1.6)	100 (0.8)	0.043	1.10 (0.98–1.22)	0.097
Obstetrics	43 (0.6)	378 (2.8)	< 0.0001	0.29 (0.22–0.38)	< 0.0001
Orthopedics	35 (0.4)	306 (2.3)	< 0.0001	0.29 (0.21–0.39)	< 0.0001
Psychiatry	14 (0.2)	407 (3.0)	< 0.0001	0.17 (0.10–0.31)	< 0.0001
Others	44 (0.6)	85 (0.6)	0.001	0.88 (0.72–1.07)	0.205

aOR, adjusted odd ratio; CI, confidence interval; ICU, intensive care unit; IQR, interquartile range; HIV, human immunodeficiency virus; SCT, stem cell transplant; SIRS, systemic inflammatory response syndrome; WBC, white blood count

^α^Generalized Estimating Equations using the REPEATED statement on PROC GENMOD was utilized for multivariable analysis

### Predictors for blood-culturing ordering practices among hypothermic patients

As there is limited knowledge regarding utilization pattern of blood cultures among hospitalized patients with hypothermia, we performed a subset analysis in this group. The results of this analysis are summarized in eTable 3. In the subset of hospitalized patients presenting with hypothermia, hospital onset hypothermia (aOR 0.49, 95% CI 0.42 to 0.57) was less likely associated with the blood culture ordering; whereas factors associated with blood culturing included presence of SIRS (aOR 2.31, 95% CI 1.67 to 2.31), SIRS plus hypotension (aOR 2.63, 95% CI 1.92 to 3.60), hypothermia ≤ 35℃ (aOR 1.49, 95% CI 1.28 to 1.73), central venous catheter (aOR 1.69, 95% CI 1.37 to 2.08), indwelling urinary catheter (aOR 1.41, 95% CI 1.12 to 1.76), and detection of hypothermia during night shift (aOR 1.31, 95% CI 1.14 to 1.50).

## Discussion

In this large retrospective cohort of 15,788 inpatients with 21,383 episodes of abnormal body temperature, we have two main findings. First, we established the incidence rate of inpatient febrile illness and hypothermia to be 20.8 and 13.2 episodes per 1,000 patient-days, respectively. To our knowledge, this is the first study to describe the incidence rates of both fever and hypothermia per patient. These incidence rates have not been well quantified by previous epidemiologic studies that showed a highly variable rate of fever, ranging from 2 to 29%. [1–3]

Second, we found high utilization and wide variation in blood culturing practices among febrile or hypothermic inpatients. Although fever is a common clinical variable that triggers blood culture ordering, several studies concluded that fever alone has a poor predictive value for bacteremia. [4–6] Published clinical practice guidelines also lack clear indications when blood cultures should be drawn. [14] In our study, we observed more than half of the febrile hospitalized patients had at least one associated blood culture obtained. Consistent with a previous study [5], we found < 5% of the blood cultures yielded true-positive results among this patient population. Our study also adds to the literature showing 13.2% of the hypothermic inpatients had at least associated blood cultures drawn -with a true positive rate and diagnostic yield of < 1% and 6.3%, respectively. While it is not entirely clear why hypothermic hospitalized patients have less associated blood culture ordering compared to febrile inpatients, a prior study has shown severe sepsis patients with hypothermia had a lower utilization of sepsis bundles compared to other patients with severe sepsis [19]. It is postulated that hypothermia is perceived by clinicians as being less alarming, as opposed to other more immediately recognizable signs (e.g., hypotension) as an ominous finding to the physicians to rapidly initiate intervention [20].

Notably, our multivariable analysis highlighted non-clinical factors, such as detection of an abnormal body temperature during night shift or on the weekend, to be independently associated with an increased risk for blood culturing among febrile or hypothermic inpatients. The practice of “pan-culturing” which includes indiscriminate ordering of microbiology culture of blood, urine, and sputum for the evaluation of fever in hospitalized patients is not uncommon. [21, 22] A recent survey showed that 80% of the respondents agreed that clinicians should order blood cultures reflectively in response to fever. [12] This practice of reflexive blood culture ordering was found to be more prevalent among cross-covering residents at night shift. [23, 24] Potential explanation for this observation in the survey include fewer staffing and supervision, and less familiarity with the patients during night shifts and on the weekends. [25] Our study expands on previous studies [12, 13] by providing a better understanding on how variation in shift work and day of the week may influence the clinical decision making around blood culture. Interventions targeting this group of clinicians with outlying ordering behavior may reduce inappropriate and unnecessary blood culture testing.

In our study, adult inpatients with SIRS and SIRS plus hypotension were more likely to have blood culture performed. Previous studies observed rising trends in blood culture usage in the emergency departments among patients hospitalized for community acquired pneumonia following the implementation of core measures by Centers for Medicare and Medicaid Services (CMS) and the Joint Commission on Accreditation of Healthcare Organizations, which mandated to obtain blood cultures prior to antibiotic administration. [26, 27] It is possible that similar national core quality measures by CMS which mandate blood cultures to be obtained for Severe Sepsis and Septic Shock Early Management (SEP-1) [28] may have driven an increased use of blood cultures among inpatients with SIRS or SIRS plus hypotension as observed in our study. However, this causal relationship or temporal association was not assessed in our analysis. Further studies are needed to define how national guidelines affect blood culture utilization pattern.

Presence of central venous catheter was another significant independent predictor for blood culture ordering in hospitalized patients with abnormal body temperature. One possible explanation for this observation is that current clinical practice guidelines by Infectious Disease Society of America (IDSA) endorses drawing blood cultures in febrile patients with central venous catheter and suspected catheter-related bloodstream infection (CRBSI). [29] Although fever is a common clinical manifestation for CRBSI, other infectious and non-infectious etiologies for fever should be investigated. The decision to obtain blood cultures should only be made if patient has a high pretest probability of bacteremia after the review of relevant clinical history and appropriate physical exam. [30]

Lastly, we found febrile or hypothermic inpatients on SCT / medical oncology services were significantly associated with blood culture ordering. Several factors may explain this finding. In our study, the proportion of patients with longer length of hospital stays was significantly higher among adult inpatients in SCT / medical oncology services compared to other services (21 days [IQR, 9 to 35 days] vs. 12 days [6 to 26 days]; p < .001). A previous study has shown a higher resource utilization in patients who had a longer hospital stay. [31] Current clinical practice guidelines by American Society of Clinical Oncology and IDSA also recommend obtaining two sets of blood culture in patients with febrile neutropenia. [32, 33] These combined factors may drive higher blood culture testing patterns seen in this patient population, however, this was not specifically assessed in our study.

Our study has limitations due to the retrospective study design and performance in a single academic hospital. Blood culture utilization patterns observed in our institution may not be generalizable to other institutions. We evaluated blood culturing practices among febrile and hypothermic patient (high-risk) population only and did not include blood cultures obtained for adult inpatients with a normal body temperature. The definition of hypothermia, euthermia/ normothermia, and hyperthermia may vary, based on the methods of body temperature measurement. We also did not evaluate whether patients received any antimicrobial treatment at the time of blood culture and the impact it may have had on the decision to obtain cultures. We do not have information on patient home temperature monitoring and receipt of antipyretic medications prior to admission. While we were able to evaluate the role of extreme hyperthermia (≥ 39℃), we could not evaluate the independent role of extreme hypothermia due to small sample size. Finally, our study was also limited by the absence of detailed chart review so we could not determine test indication (e.g., follow up blood cultures after initial positive blood culture), characteristics of provider ordering the culture (e.g., level of training), and impact of blood culture on antibiotic decision making. However, to date, this is the largest and well-characterized cohort of adult inpatients with fever and hypothermia and our study identified factors that may influence blood culture ordering practices. To our knowledge, our study is the first to address the clinical question regarding blood culture utilization for hospitalized patients with hypothermia.

In summary, our study suggests decision making around blood cultures among hospitalized patients with fever or hypothermia is multifactorial; both clinical and non-clinical factors. Our findings highlight the need for clinical practice guidelines to address clear and evidence-based indications for blood culture among patients with abnormal body temperature to avoid unnecessary and inappropriate testing. Future studies should look into practices over weekends/ off hours and its impact on blood cultures in detail, and develop an algorithm to identify high risk groups. Future interventions targeting improvement in blood culture ordering behaviors should be also considered.

## Electronic supplementary material

Below is the link to the electronic supplementary material.

Supplementary Material 1

## Data Availability

The datasets generated and/or analyzed during the current study are maintained by our institution under a secure server and are not publicly available as they contain protected health information. The data that support the findings of this study are available from the corresponding author upon reasonable request.
