# Supplementary material for: Blood culture utilization practices among febrile and/or hypothermic inpatients
Source: BMC Infect Dis. 2022 Oct 10;22:779. doi: 10.1186/s12879-022-07748-x (PMC9552399; doi:10.1186/s12879-022-07748-x)
Supplement: Supplementary file 1 — Supplementary Material 1 [file 12879_2022_7748_MOESM1_ESM.docx]

**Supplementary Material**

**Table of Contents**

Page 2- eTable 1. International classification of diseases (ICD), 10th revision codes used for defining comorbid conditions.

Page 3. eTable 2. Microbiology data for 672 positive blood cultures

Page 4. eTable 3. Comparison of factors for blood culture ordering among hypothermic adult inpatients.

Kap Sum Foong MD^1,2^, Satish Munigala MBBS MPH^2^, Stephanie Kern-Allely BS^3^, David K Warren MD MPH^2^

eTable 1. International classification of diseases (ICD), 10th revision codes used for defining comorbid conditions.

| **Comorbidity** | **ICD 10 codes** |
| --- | --- |
| Congestive heart failure | I099, I110, I130, I132, I255, I420, I425, I426, I427, I428, I429, I43, I50, P290 |
| Chronic lung disease | I278, I279, J40, J41, J42, J43, J44, J45, J46, J47, J60, J61, J62, J63, J64, J65, J66, J67, J684, J701, J703 |
| Diabetes mellitus | E100, E101, E109, E110, E111, E119, E120, E121, E129, E130, E131, E139, E140, E141, E149, E102, E103, E104, E105, E106, E107, E108, E112, E113, E114, E115, E116, E117, E118, E122, E123, E124, E125, E126, E127, E128, E132, E133, E134, E135, E136, E137, E138, E142, E143, E144, E145, E146, E147, E148 |
| Chronic liver failure or liver disease | B18, I85, I864, I982, K70, K711, K713, K714, K715, K717, K72, K73, K74, K760, K762, K763, K764, K765, K766, K767, K768, K769, Z944 |
| End stage renal disease | I120, I131, N18, N19, N250, Z490, Z491, Z492, Z940, Z992 |
| Malignancy (solid tumor without metastasis and metastatic cancer) | C77, C78, C79, C80, C00, C01, C02, C03, C04, C05, C06, C07, C08, C09, C10, C11, C12, C13, C14, C15, C16, C17, C18, C19, C20, C21, C22, C23, C24, C25, C26, C30, C31, C32, C33, C34, C37, C38, C39, C40, C41, C43, C45, C46, C47, C48, C49, C50, C51, C52, C53, C54, C55, C56, C57, C58, C60, C61, C62, C63, C64, C65, C66, C67, C68, C69, C70, C71, C72, C73, C74, C75, C76, C97 |
| HIV infection | B20, B21, B22, B24 |

HIV, human immunodeficiency virus.

eTable 2. Microbiology data for 672 positive blood cultures

|  | Organisms isolated^α^  n= 754; n (%) | Organisms isolated from hyperthermia patients  n=679; n (%) | Organisms isolated from hypothermia patients  n=75; n (%) | *p*-value |
| --- | --- | --- | --- | --- |
| *Staphylococcus aureus* | 166 (22.0) | 156 (23.0) | 10 (13.3) | 0.057 |
| Coagulase negative *Staphylococcus*^β^ | 80 (10.6) | 66 (9.7) | 14 (18.7) | 0.027 |
| *Escherichia coli* | 69 (9.1) | 63 (9.3) | 6 (8.0) | 0.835 |
| *Candida* spp | 53 (7.0) | 44 (6.5) | 9 (12.0) | 0.092 |
| *Staphylococcus epidermidis* | 43 (5.7) | 40 (5.9) | 3 (4.0) | 0.791 |
| *Enterococcus faecium* | 40 (5.3) | 34 (5.0) | 6 (8.0) | 0.274 |
| *Klebsiella pneumoniae* | 39 (5.2) | 34 (5.0) | 5 (6.7) | 0.579 |
| *Enterococcus faecalis* | 37 (4.9) | 34 (5.0) | 3 (4.0) | 1.00 |
| *Pseudomonas aeruginosa* | 37 (4.9) | 31 (4.6) | 6 (8.0) | 0.251 |
| *Streptococcus* spp | 36 (4.9) | 36 (5.3) | 0 (0.0) | 0.041 |
| *Enterobacter cloacae* complex | 26 (3.4) | 24 (3.5) | 2 (2.) | 1.00 |
| Others | 128 (17.0) | 117 (17.2) | 11 (14.6) |  |

Spp, species.

^α^9.7% of the positive blood cultures were polymicrobials.

^β^Excluded *Staphylococcus epidermidis.*

eTable 3. Comparison of factors for blood culture ordering among hypothermic adult inpatients.

| Variable | Blood culture performed  n= 1092; n (%) | No blood  culture performed  n= 7198; n (%) | Univariate analysis | Multivariable analysis ^α^ | |
| --- | --- | --- | --- | --- | --- |
|  |  |  | ***p* value** | **aOR**  **(95% CI)** | ***p* value** |
| Age in years, median (IQR) | 62 (52-71) | 62 (50-71) | 0.88 | - |  |
| Gender |  |  | 0.894 | - |  |
| Male | 601 (55.0) | 3946 (54.8) |  |  |  |
| Female | 491 (45.0) | 3252 (45.2) |  |  |  |
| Race |  |  | <.0001 |  |  |
| White | 631 (57.8) | 4779 (66.4) |  | Reference |  |
| Black or others | 461 (42.2) | 2419 (33.6) |  | 1.45 (1.26-1.67) | <.0001 |
| Extreme hypothermia (≤35⁰C) | 361 (33.1) | 1655 (23.0) | <.0001 | 1.49 (1.28-1.73) | <.0001 |
| Hospital-onset hypothermia | 510 (46.7) | 4397 (61.1) |  | 0.49 (0.42-0.57) | <.0001 |
| Comorbidities |  |  |  |  |  |
| Congestive heart failure | 447 (40.9) | 2625 (36.5) | 0.004 | - | - |
| Chronic lung disease | 302 (27.7) | 1975 (27.4) | 0.838 | - | - |
| Diabetes mellitus | 376 (34.4) | 2208 (30.7) | 0.013 | 1.17 (1.01-1.35) | 0.035 |
| Chronic liver failure | 371 (34.0) | 1374 (19.1) | <.0001 | 1.53 (1.32-1.78) | <.0001 |
| End stage renal disease | 383 (35.1) | 2253 (31.3) | 0.01 | - | - |
| Malignancy | 143 (13.1) | 988 (13.7) | 0.572 | - | - |
| HIV infection | 10 (0.9) | 68 (0.9) | 0.933 | - | - |
| SIRS status |  |  |  |  |  |
| No SIRS | 70 (6.4) | 2140 (29.7) | Reference | Reference |  |
| SIRS alone | 161 (14.7) | 1119 (15.6) | <.0001 | 2.31 (1.67-3.21) | <.0001 |
| SIRS plus hypotension | 861 (78.9) | 3939 (54.7) | <.0001 | 2.63 (1.92-3.60) | <.0001 |
| WBC |  |  |  |  |  |
| Normal or not done | 378 (34.6) | 3042 (42.3) | Reference | - | - |
| <4,000 /mm^3^ | 126 (11.5) | 786 (10.9) | 0.021 |  |  |
| >12,000 /mm^3^ | 588 (53.9) | 3370 (46.8) | <.0001 |  |  |
| Shift work during detection of abnormal body temperature |  |  | <.0001 |  |  |
| Day (8am to 8pm) | 576 (52.7) | 4494 (62.4) |  | Reference |  |
| Night (8pm to 8am of the  following day) | 516 (47.3) | 2704 (37.6) |  | 1.31 (1.14-1.50) | <.0001 |
| Weekend (Saturday & Sunday) | 282 (25.8) | 1631 (22.7) | 0.021 | - | - |
| Mechanical ventilation | 738 (67.6) | 3249 (45.1) | <.0001 | - | - |
| Indwelling urinary catheter | 952 (87.2) | 4999 (69.5) | <.0001 | 1.41 (1.12-1.76) | 0.003 |
| Central venous catheter | 917 (84.0) | 4594 (63.8) | <.0001 | 1.69 (1.37-2.08) | <.0001 |
| Department during detection of hypothermia |  |  |  |  |  |
| Emergency department | 102 (9.3) | 290 (4.0) | <.0001 | 1.81 (1.52-2.16) | <.0001 |
| General medicine | 85 (7.8) | 727 (10.1) | Reference | Reference |  |
| Surgery | 25 (2.3) | 791 (11.0) | <.0001 | 0.28 (0.17-0.44) | <.0001 |
| ICU | 804 (73.6) | 3962 (55.0) | <.0001 | 0.67 (0.50-0.88) | 0.004 |
| SCT / medical oncology | 53 (4.9) | 272 (3.8) | 0.007 | 1.37 (0.92-2.05) | 0.119 |
| Cardiology | 10 (0.9) | 206 (2.9) | 0.011 | 0.33 (0.16-0.65) | 0.002 |
| Neurology / neurosurgery | 8 (0.7) | 256 (3.6) | <.0001 | 0.38 (0.18-0.80) | <.0001 |
| Gynecologic oncology | 2 (0.2) | 33 (0.5) | 0.373 | 0.77 (0.18-3.34) | 0.722 |
| Obstetrics | 2 (0.2) | 139 (1.9) | 0.004 | 0.25 (0.06-1.06) | 0.06 |
| Orthopedics | 0 (0.0) | 122 (1.7) | - | - | - |
| Psychiatry | 0 (0.0) | 341 (4.7) | 0.958 | - | - |
| Others | 1 (0.1) | 59 (0.8) | 0.057 | 0.15 (0.02-1.14) | 0.067 |

aOR, adjusted odd ratio; CI, confidence interval; ICU, intensive care unit; IQR, interquartile range; HIV, human immunodeficiency virus; SCT, stem cell transplant; SIRS, systemic inflammatory response syndrome; WBC, white blood count.

^α^Forward step-wise logistic regression analysis with *p*=0.20 for entry and *p*=0.15 for stay was utilized for multivariate analysis.
